# Effects of Long-Term Treatment with a Blend of Highly Purified Olive Secoiridoids on Cognition and Brain ATP Levels in Aged NMRI Mice

**DOI:** 10.1155/2018/4070935

**Published:** 2018-10-30

**Authors:** Martina Reutzel, Rekha Grewal, Carmina Silaidos, Jens Zotzel, Stefan Marx, Joachim Tretzel, Gunter P. Eckert

**Affiliations:** ^1^Institute of Nutritional Sciences, Laboratory for Nutrition in Prevention and Therapy, Justus-Liebig-University of Giessen, Biomedical Research Center Seltersberg (BFS), Schubertstr. 81, 35390 Giessen, Germany; ^2^N-Zyme BioTec GmbH, Haasstraße 9, 64293 Darmstadt, Germany

## Abstract

Aging represents a major risk factor for developing neurodegenerative diseases such as Alzheimer's disease (AD). As components of the Mediterranean diet, olive polyphenols may play a crucial role in the prevention of AD. Since mitochondrial dysfunction acts as a final pathway in both brain aging and AD, respectively, the effects of a mixture of highly purified olive secoiridoids were tested on cognition and ATP levels in a commonly used mouse model for brain aging. Over 6 months, female NMRI mice (12 months of age) were fed with a blend containing highly purified olive secoiridoids (POS) including oleuropein, hydroxytyrosol and oleurosid standardized for 50 mg oleuropein/kg diet (equivalent to 13.75 mg POS/kg b.w.) or the study diet without POS as control. Mice aged 3 months served as young controls. Behavioral tests showed deficits in cognition in aged mice. Levels of ATP and mRNA levels of NADH-reductase, cytochrome-c-oxidase, and citrate synthase were significantly reduced in the brains of aged mice indicating mitochondrial dysfunction. Moreover, gene expression of Sirt1, CREB, Gap43, and GPx-1 was significantly reduced in the brain tissue of aged mice. POS-fed mice showed improved spatial working memory. Furthermore, POS restored brain ATP levels in aged mice which were significantly increased. Our results show that a diet rich in purified olive polyphenols has positive long-term effects on cognition and energy metabolism in the brain of aged mice.

## 1. Introduction

Aging represents one of the major risk factors for developing neurodegenerative diseases such as Alzheimer's disease (AD). Currently, five million Americans are suffering from dementia, and by 2050, there will be one new case every 66 seconds [1]. The multifactorial pathology makes it difficult to develop feasible therapies, and current approved drugs attenuate symptoms but do not cure the disease. Research into AD also had several failures in terms of developing disease-modifying therapies [2]. Since AD starts many years before the first symptoms occur, new scientific approaches focus on early stages, which are discussed to be important in aging and the onset of AD. In this sense, there is growing interest in dietary patterns, stimulation of the brain, and physical activity as potential modifiable risk factors [3–6]. It has been shown that adherence to a Mediterranean diet (MedDiet) pattern significantly reduces the risk of AD [7, 8], which has been confirmed and recently summarized in reviews and meta-analysis [9–11].

One important component of the MedDiet is a high consumption of extra virgin olive oils (EVOO) [12], and a combination of MedDiet and EVOO seems to better improve cognitive function including improved performance in visual and verbal memory domains [13–15].

EVOO contains several different polyphenols [12] including secoiridoid derivatives, phenolic alcohols, and lignans as well as flavonoids which seem to have neuroprotective properties on the aging process [16–18]. Hydroxytyrosol and oleuropein are two of the main antioxidative compounds present in olives [19–22] providing neuroprotection [22–26]. Thus, olive polyphenols are proposed as new promising agents to combat aging-associated neurodegeneration [27].

**Figure 1:**
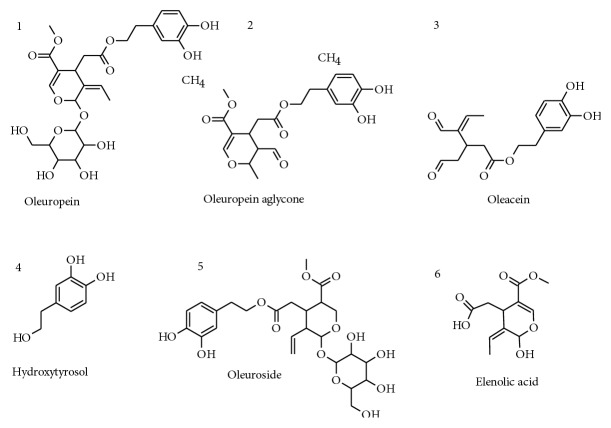
Secoiridoid derivatives (1–5) and their degradation products are the predominant phenolic compounds present in EVOO and in the tested POS. Secoiridoids are characterized by the presence in their molecules of elenolic acid (6) or its derivatives.

Using a recently developed technology, olive polyphenols were isolated and highly purified from olive leaves that represent a rich source of bioactive ingredients [28]. We investigated the effects of a mixture of six highly purified secoiridoid polyphenols (Figure 1) on cognition and brain energy metabolism in aged NMRI mice. Expression of genes involved in longevity, mitochondrial biogenesis and function, synaptic plasticity, and antioxidative properties was determined to elaborate molecular mechanisms. Effects on ATP levels were confirmed in neuronal SH-SY5Y cells.

## 2. Materials and Methods

### 2.1. Cell Culture

SH-SY5Y cells were cultured in Dulbecco's Modified Eagle Medium supplemented with 10% heat-inactivated fetal calf serum, 0.3 mg/mL hygromycin, 60 units/mL penicillin, 60 *μ*g/mL streptomycin, 4500 mg/L D-glucose, MEM Vitamin solution, MEM Nonessential Amino Acids, and 1 mM sodium pyruvate at 37°C in a humidified incubator containing 5% CO_2_.

Two days prior to incubation, cells were seeded into 96-well plates (20,000 cells/well). Cells were incubated with the respective POS concentrations (0.001 nM–10 *μ*M dissolved in DMEM) for 24 h (basal ATP levels) or preincubated with POS for 1 h and insulted with rotenone (25 *μ*M) for 24 h (ATP levels after insult). The emitted light is linear to the ATP (Lonza, Switzerland) concentration and was measured with a VICTOR*™* X2 Multilabel Plate Reader (Perkin Elmer).

### 2.2. Animals and Treatment

Female NMRI (Navar Medical Research Institute) mice, a commonly used aging model, were purchased from Charles River (Sulzbach, Germany) and were housed according to the German guidelines for animal care with access to water and food ad libitum. Mice were maintained on a 12 h light/dark cycle until they reached the age of 12 months. Young (3 months old) NMRI mice served as the control group. Mice were fed with a well-proven C1000 standardized diet (Altromin, Lage, Germany), C1000 containing vitamin A (2500 IU/kg), vitamin E (20 mg/kg), and selenium (150 mcg/kg) [29, 30]. The verum group received the identical diet supplemented with a blend of highly purified secoiridoid polyphenols for 6 months. Based on the average food consumption, a daily intake of 13.75 mg POS/kg b.w. was calculated. Feeding studies in mice reporting biological effects applied olive polyphenols in a dose range of 1 to 10 mg/kg b.w. [26, 31–33]. The estimated daily POS dose (13.75 mg/kg b.w.) in the current study equals a single human dose of approximately 1.1 mg/kg b.w/day [34]. The feeding period of the young control mice started 3 months later than the feeding period of aged mice to ensure that both of them end at the same time point. Behavioral testing was performed before the starting points and at the end of the feeding period. On the basis of behavioral testing at the beginning, mice were divided into 2 groups of the same performance level. Mice were killed by cervical dislocation and decapitation. Brains were quickly dissected on ice after the removal of the cerebellum, the brain stem, and the olfactory bulb. All experiments were carried out by individuals with appropriate training and experience according to the requirements of the Federation of European Laboratory Animal Science Associations and the European Communities Council Directive (Directive 2010/63/EU). Experiments were approved by the regional authority (Regierungspraesidium Darmstadt; #V54–19 c 20/15–FU/1062).

### 2.3. Composition of the POS

The POS was provided from N-Zyme Biotec GmbH, Darmstadt, Germany. The composition is listed in Table 1.

### 2.4. Passive Avoidance Test

The test was conducted using a passive avoidance step-through system (cat. no. 40533/mice Ugo Basile, Gemonio, Italy) and a protocol similar to the protocol published by Shiga et al. [35]. On the first day of the experiment, the mouse was put into the light chamber (light intensity of 1350 lux). After 30 s, the door toward the dark chamber was opened, and time till entering the dark chamber was measured. In the dark chamber, the mouse received an electric shock (0.5 mA, 1 s duration). The test was stopped if the mouse did not enter the dark chamber after 180 s. The test was repeated after 24 h with the door opening towards the dark chamber after only 5 s. Again, time till entering the dark chamber was recorded. This time no electric shock was applied after crossing the door. The test was stopped after 300 s.

### 2.5. One-Trial Y-Maze Test

One-trial Y-Maze test was conducted using a custom-made Y-Maze (material: polyvinyl chloride, length of arms: 36 cm, height of arms: 7 cm, width of arms: 5 cm, and angle between arms: 120°). At the beginning of the test, the mouse was put into one of the three arms of the Y-Maze and the sequence of the entries was recorded for 5 min. Spontaneous alternation was determined using the formula (number of alternations/number of entries)/2 [36].

### 2.6. Preparation of Dissociated Brain Cells for the Measurement of the Mitochondrial Membrane Potential and Determination of ATP Level

One hemisphere was used to prepare dissociated brain cells (DBCs) for ex vivo studies according to the method of [30]. DBCs were resuspended in 4.5 mL DMEM without supplements. For the measurement of ATP levels, DBCs were seeded in 50 *μ*L aliquots into a 96-well plate. Cells were incubated for 3 h in a humidified incubator (5% CO_2_). Respectively, 6 wells were incubated for 3 h with sodium nitroprusside (0.5 mM for ATP measurement) in DMEM. The remaining cell suspension was reserved for protein determination (stored at −80°C).

### 2.7. Protein Quantification

Protein content was determined using Pierce^TM^ Protein Assay Kit (Thermo Fisher Scientific, Waltham, MA, USA). Instructions were followed as given by the manufacturer.

### 2.8. Glutathione Peroxidase Activity

Glutathione peroxidase activity was determined using a commercially available assay kit (kit number: ab102530; Abcam Plc., Cambridge, UK). 50 mg of frozen brain tissue was used as described in the manufacturer's manual. Glutathione peroxidase generates GSSG from GSH during H_2_O_2_ reduction, and the generated GSSG is reduced back to GSH by glutathione reductase during consumption of NADPH. The reduction of NADPH is proportional to glutathione peroxidase activity; thus, it can be measured calorimetrically at 340 nm.

### 2.9. Citrate Synthase Activity

Citrate synthase activity was determined photometrically in isolated brain mitochondria as recently described in Hagl et al. [37].

### 2.10. Isolation of Brain Mitochondria and Measurement of Complex I and IV Activities

Half a brain hemisphere (the frontal part) was used to isolate brain mitochondria. The protocol is described in Hagl et al. [37]. The pellet obtained from the last centrifugation step was dissolved in 250 *μ*L MIRO5. 80 *μ*L of the resulting cell suspension was injected into the Oxygraph 2k-chamber. A complex protocol (elaborated by Prof. Dr. Erich Gnaiger) was used to investigate the function of the respiratory chain complexes. The capacity of the oxidative phosphorylation (OXPHOS) was determined using complex I-related substrates pyruvate (5 mM) and malate (2 mM) and ADP (2 mM) followed by the addition of succinate (10 mM). Mitochondrial integrity was measured by the addition of cyctochrome c (10 *μ*M). Oligomycin (2 *μ*g/mL) was added to determine leak respiration (leak (omy)), and afterwards, uncoupling was achieved by carbonyl cyanide p-(trifluoromethoxy) phenyl-hydrazone (FCCP, injected stepwise up to 1–1.5 *μ*M). Complex II respiration was measured after the addition of rotenone (0.5 *μ*M). Complex III inhibition was achieved by the addition of antimycin A (2.5 *μ*M) and was subtracted from all respiratory parameters. COX activity was measured after ROX determination by applying 0.5 mM tetramethylphenylenediamine (TMPD) as an artificial substrate of complex IV and 2 mM ascorbate to keep TMPD in the reduced state. Autoxidation rate was determined after the addition of sodium azide (>100 mM), and COX respiration was additionally corrected for autoxidation.

### 2.11. Gene Expression Analysis by Quantitative Real-Time PCR (qRT-PCR)

Total RNA was isolated using the RNeasy Mini Kit (Qiagen, Hilden, Germany) according to the manufacturer's instructions using ~20 mg RNAlater stabilized samples (Qiagen, Hilden, Germany). RNA was quantified measuring the absorbance at 260 and 280 nm using the NanoDrop™ 2000c spectrometer (Thermo Fisher Scientific, Waltham, MA, USA). RNA purity was assessed using the ratio of absorbance 260/280 and 260/230. To remove residual genomic DNA, samples were treated with a TURBO DNA-free™ kit according to the manufacturer's instructions (Thermo Fisher Scientific, Waltham, MA, USA). Complementary DNA was synthesized from 250 ng total RNA using the iScript cDNA Synthesis Kit (Bio-Rad, Munich, Germany) according to the manufacturer's instructions and was stored at −80°C. qRT-PCR was conducted using a CfX 96 Connect™ system (Bio-Rad, Munich, Germany). Oligonucleotide primer sequences, primer concentrations, and product sizes are listed in Table 2. All primers were received from Biomol. cDNA for qRT-PCR was diluted 1 : 10 with RNase-free water (Qiagen, Hilden, Germany), and all samples were performed in triplicate. PCR cycling conditions were an initial denaturation at 95°C for 3 min, followed by 45 cycles of 95°C for 10 s, 58°C for 45 s, and 72°C for 29 s. Gene expression was analyzed using the −(2∆∆C_q_) method using BioRad CfX manager software and was normalized to the expression levels of beta 2 microglobulin (B2M) and phosphoglycerate kinase 1 (PGK1).

### 2.12. Statistics

Unless otherwise stated, values are presented as mean ± standard error of the mean (SEM). Statistical analyses were performed by applying one-way ANOVA with Bonferroni's multiple comparison posttest (Prism 7.0 GraphPad Software, San Diego, CA, USA). Statistical significance was defined for *p* values of <0.05.

## 3. Results

Female NMRI mice were fed over 6 months with a standardized pelleted diet (aged control) or diet supplemented with a blend of highly purified secoiridoid polyphenols (13.75 mg POS/kg b.w.) (aged + POS) for 6 months. Young control mice (young control) received a standardized pelleted diet for 3 months. There was no significant difference in body weight and life span between the aged control and the aged intervention group mice. At the end of the feeding period, cognitive function and brain mitochondrial function were assessed.

### 3.1. Survival

**Figure 2:**
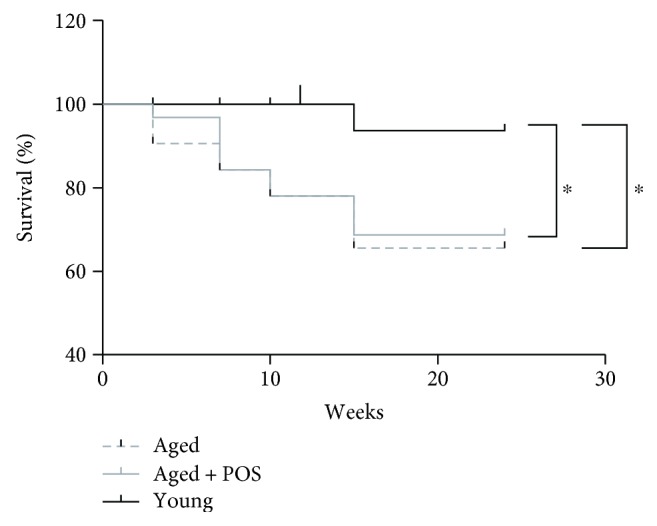
Survival rates of NMRI mice after feeding with or without POS. Aged (12 months old) mice were fed with a standardized pelleted diet (aged) or pelleted diet containing POS (13.75 mg POS/kg b.w., aged + POS) for 6 months. As further control, young mice (3 months old, starting point of the analysis: 12 weeks) were fed with a pelleted standard diet for 3 months (young); *n* = 15 − 27; mean without SEM; log-rank (Mantel-cox) test; ^∗^ *P* < 0.05.

Survival rates of young and aged control mice were 93 and 66% (*P* < 0.05), while the survival rate of aged mice administrated with POS was 69% (*P* < 0.05). Thus, POS treatment did not increase the survival rate over a 6-month feeding period (Figure 2).

#### 3.1.1. Behavioral Testing

**Figure 3:**
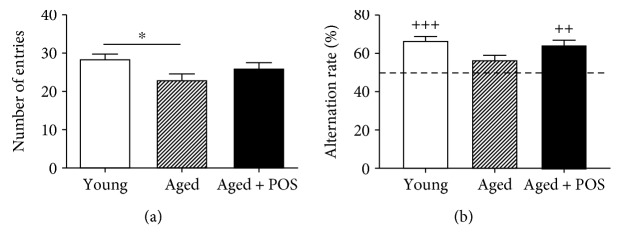
Y-Maze spontaneous alternation of young, aged, and POS-treated mice (13.75 mg/kg b.w.) during a 5 min period time of testing. Number of entries (a) and alternation rate (b); *n* = 16 mean ± SEM, one-way ANOVA with Bonferroni posttest; ^∗^ *P* < 0.05; ^∗∗^ *P* < 0.01; ^∗∗∗^ *P* < 0.001. Alternation rate (c) was compared to a theoretical value of 50% using a univariate *t*-test with ^++^ *P* < 0.01 and ^+++^ *P* < 0.001.

In the Y-Maze test, aged control mice showed a significantly decreased alternation rate (56.7 ± 2%) and number of entries (23 ± 1.5) during a 5 min testing phase compared to young controls (Figure 3(b)). POS administration for 6 months significantly increased the alternation rate (64 ± 2%) and slightly but not significantly increased the number of entries (26 ± 1) (Figure 3(a)).

**Figure 4:**
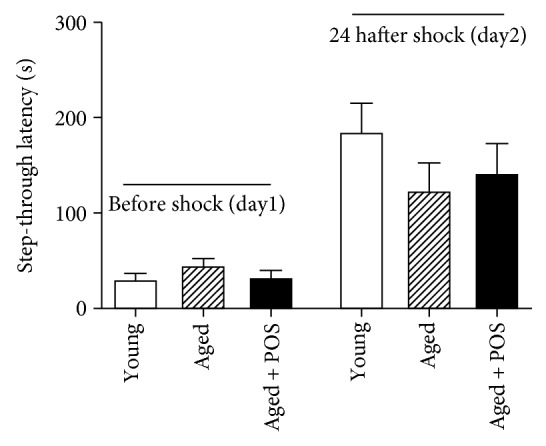
Passive avoidance test with young, aged, and long-term POS-treated mice. On day one, mice receive an electric shock (0.5 mA) and time is recorded when the mouse needs to enter into the dark chamber; 24 h after the first testing period, the test is repeated and time is recorded when the mouse needs to reenter the dark chamber; *n* = 16; mean ± SEM.

On day one, aged control mice showed a slightly but not significant longer latency time to enter into the dark chamber (45 ± 7 s) compared to young control animals (31 ± 6 s) in the passive avoidance test. POS-treated mice showed almost the same latency time as young mice (33 ± 7 s). On day two, aged mice showed a numerically shorter time to reenter the dark chamber (123 ± 29 s) compared to young control mice (185 ± 29 s). POS treatment for 6 months led to a slightly but not significant increased step-through latency time (142 ± 31 s) (Figure 4).

#### 3.1.2. Effect of Long-Term POS Treatment on Brain ATP Levels

**Figure 5:**
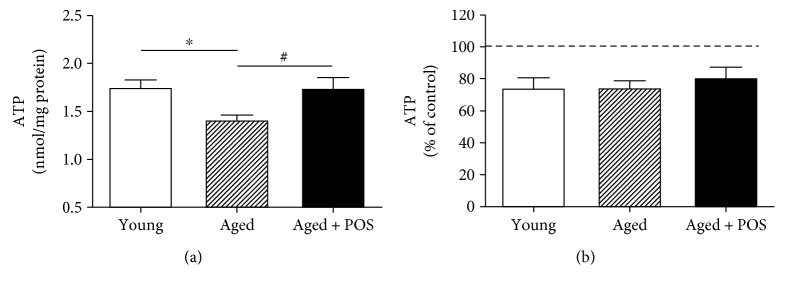
Basal ATP level (a) and ATP concentrations after insult with sodium nitroprusside (SNP, 3 h, 0.1 mM) (b) of dissociated brain cells (DBCs) from young, aged, and POS-treated mice; basal ATP concentrations served as control for normalization in (b); *n* = 10; mean ± SEM; one-way ANOVA with Bonferroni posttest; ^∗^ *P* < 0.05 vs. young; ^#^one-way ANOVA aged vs. aged + POS with ^#^ *P* < 0.05.

**Figure 6:**
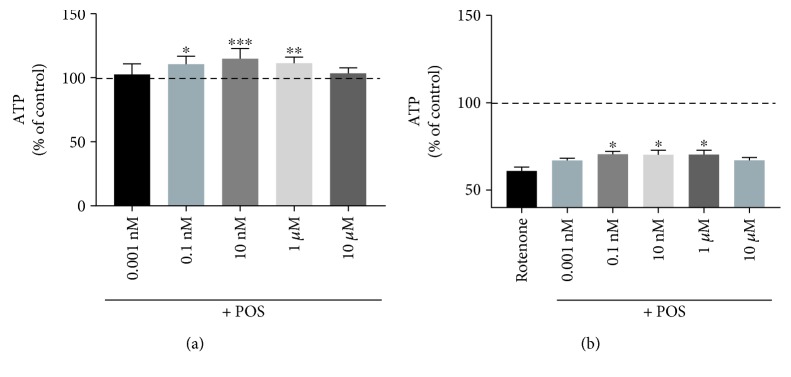
Basal ATP level (a) and ATP concentrations after incubation for 24 h in SY5Y-Mock cells with POS of different concentrations (0.001 nM–10 *μ*M) and insult with rotenone 250 nm (b) in SH-SY5Y control cells; basal ATP concentrations served as control, *n* = 7, mean ± SEM, one-way ANOVA with Bonferroni posttest; ^∗^ *P* < 0.05, ^∗∗^ *P* < 0.01, ^∗∗∗^ *P* < 0.001.

Basal ATP levels were measured in dissociated brain cells (DBCs) of young, aged, and POS-treated mice. Aged control mice showed significantly lower ATP level (1.41 ± 0.05 nmol/mg protein) in contrast to young animals (1.75 ± 0.01 nmol/mg protein) which were restored after long-term treatment with POS (1.74 ± 0.1 nmol/mg protein; Figure 5(a)). Furthermore, DBCs were incubated for 3 h with sodium nitroprusside (SNP) to examine the resistance against nitrosative stress. However, no differences were detected after SNP incubation between young (74.4 ± 2.2%), aged (74.7 ± 1.3%), and POS-treated aged mice (81.3 ± 2.2%). Long-term POS treatment resulted in a slight increase of ATP concentrations after SNP insult which did not reach a level of significance (Figure 5(b)). To confirm the effects of POS on ATP levels in vitro, SH-SY5Y cells were incubated with different concentrations of POS. A POS concentration of already 0.1 nM significantly enhanced basal ATP levels (Figure 6(a)). At this concentration, SH-SY5Y cells were also protected from nitrosative stress induced by SNP (Figure 6(b) and Table 3).

Expression of genes involved in longevity, mitochondrial biogenesis and function, synaptic plasticity, and antioxidative properties was determined in young, aged, and POS-treated mice to elaborate molecular mechanisms. All considered genes showed significantly decreased mRNA levels after aging with the exception of SOD2. Long-term POS treatment did not show any significant effects on mRNA expression levels between aged and POS-treated mice (Table 4).

### 3.2. Activities of Complex I, Complex IV, GPx-1, and CS

In comparison to young control animals, activities of the respiratory chain complexes I and IV and CS activity were unaffected during the aging process and after long-term POS treatment in isolated brain mitochondria. Furthermore, we measured the activities of the antioxidative enzyme GPx-1 in total brain homogenate. The activity of GPx-1 was numerically but not significantly reduced in aged control animals compared to young mice (Table 5).

## 4. Discussion

In the current study, the effects of long-term feeding of a blend with highly purified olive secoiridoids on cognition and brain ATP levels were tested in aged female NMRI mice. This strain represents a well-established model for aging studies [30, 38–40]. The results show that a diet rich in purified olive polyphenols has positive long-term effects on cognition and energy metabolism in the brain of aged mice.

### 4.1. Cognitive Performance in Aged and POS-Treated NMRI Mice

Aged NMRI mice showed deficits in spatial working memory and mobility which is in agreement with earlier studies [30, 38, 41]. Our findings indicated beneficial effects of POS on spatial learning memory and mobility. In agreement with our findings, administration of olive polyphenols has been associated with the improvement of cognitive functions [26, 42, 43].

Pitozzi et al. investigated the effects of long-term dietary administration of EVOO rich in polyphenols in aged C57Bl/6J mice [43]. Comparable to our study, mice were fed from middle age to senescence (total polyphenol dose/day of 6 mg/kg b.w.), and results showed improved contextual memory and prevention of the age-related impairment in motor coordination [43]. EVOO containing different concentrations of polyphenols (e.g., tyrosol, hydroxytyrosol, verbascoside, and oleuropein di-aldehyde) induced similar beneficial effects at a comparable dose as pure oleuropein [42]. Synaptophysin 1 (Syp1) and growth-associated protein 43 (GAP43) are involved in neuronal plasticity and cognition [44, 45]. However, mice lacking SYP1 show significantly reduced learning behavior [46], and enriched environment has been reported to have positive effects on SYP1 brain levels [47]. GAP43 is a nervous tissue-specific protein and is mainly involved in development and axonal remodelling in adult brains [48]. Recently, we have reported that mRNA levels of those two proteins were significantly decreased in the brains of aged NMRI mice [49], which is confirmed by our recent data. These findings indicate less synaptic plasticity and remodelling in the brains of aged NMRI mice which might be responsible for age-related cognitive decline in memory and motor performance [50, 51].

### 4.2. Brain ATP Levels in Aged and POS-Treated NMRI Mice

The high-energy compound ATP is the key energy source in eukaryotic cells, which is mainly generated in the mitochondria by oxidative phosphorylation (OXPHOS). The mammalian OXPHOS system comprises five large complexes (including NADH oxidoreductase, succinate reductase, cytochrome c oxidoreductase, cytochrome c reductase, and ATP synthase) at the inner mitochondrial membrane [52]. In DBCs isolated from the brains of aged NMRI mice, significantly lower ATP level was determined compared to that of young controls. This finding is in agreement with earlier reports relating lower ATP levels to an impairment of CI and CIV of the OXPHOS system [30, 49, 53, 54]. Accordingly, our current data show significantly decreased expression levels of CI and CIV which also have been reported recently [30]. Long-term treatment with POS significantly improved ATP levels in the DBC of aged NMRI mice, an effect that has not been reported yet for olive polyphenols *in vivo*. POS also improved ATP levels in neuronal SH-SY5Y cells. Recent studies indicate that a mixture of 6 polyphenols (tannic acid, resveratrol, quercetin, rutin, gallic acid, and morin) is able to increase ATP levels during age-related hearing loss [55] in female rats and in the brains of a transgenic mouse model of AD [56]. In a previous study, we showed that short-term administration of hydroxytyrosol-rich olive mill waste water extract (HTRE) to NMRI mice significantly enhanced the mitochondrial membrane potential in DBC isolated from treated mice [23]. In the same study, DBCs were treated with HTRE *in vitro*, and a concentration-dependent improvement of the MMP was detected. In this study, ATP levels were not determined. However, the MMP is the driving force for complex V of the mitochondrial respiration chain (CV; F0/F1-ATPase) to generate ATP [52]. In a following study, HTRE was tested in PC12 cells and HTRE or purified hydroxytyrosol (HT) neither improved MMP nor ATP levels, indicating a different mode of action for POS and HTRE. However, both HTRE and HT protected MMP and ATP levels in PC12 cells from nitrosative stress in a concentration-dependent manner [57]. In the current study, we only detected a numerical increase of ATP levels after SNP insult in DBCs isolated from the brains of POS-treated mice. This result also indicates that POS did not provide antioxidative properties in our current study. Accordingly, POS did not improve mRNA levels and enzyme activity of GPx-1, which is involved in the endogenous response against oxidative stress in the central nervous system [58]. Sirt1 and AMPK are important players in mitochondrial biogenesis since they activate peroxisome proliferator receptor gamma coactivator 1-*α* (PGC1-*α*) [59]. PGC1*α* itself is activated by deacetylation via sirtuins (SIRT) and phosphorylation via AMP-activated protein kinases (AMPK). Furthermore, phosphorylated cAMP response element-binding protein (CREB) can induce gene expression of PGC1*α*. PGC1*α* facilitates the expression of transcription factors nuclear respiratory factor 1 (Nrf1) and mitochondrial transcription factor A (Tfam) which in turn induces mitochondrial biogenesis. POS treatment did not influence the expression levels of Sirt1 and AMPK. Additionally, we measured citrate synthase activity in isolated mitochondria which is a marker for the determination of mitochondrial content [60]. Significant lower citrate synthase (CS) mRNA expression was determined in the brains of aged and aged + POS-fed NMRI mice whereas CS in isolated mitochondria was unaffected (Table 5) indicating that other molecular mechanisms were responsible for the improvement of cognition. Possibly, the increased ATP levels and the resulting improvement of cognition are a consequence of an enhanced glycolysis which supports important functions such as neuroprotection and dramatically decreases with age [61]. Typically, glucose-6-phosphatase converts glucose into pyruvate which generates two molecules of ATP. Thus, future studies should determine the levels of glucose, lactate, glucose-6-phosphatase, and pyruvate to confirm this hypothesis.

## 5. Conclusion

Long-term feeding of a blend containing highly purified secoiridoid polyphenols (POS) provided beneficial effects on spatial working memory and motor coordination which were probably mediated by the increased ATP brain levels. Therefore, POS might represent a suitable nutraceutical for age-related cognitive decline.

## Figures and Tables

**Table 1:** Composition of the highly purified secoiridoid-rich extract (POS), manufactured by N-Zyme Biotec GmbH, Darmstadt, Germany.

Secoiridoid derivative	Content (%)
Oleuropein aglycone	36.7
Hydroxytyrosol	24.7
Oleacein	14.6
Elenolic acid derivatives	10.5
Oleuropein	7.2
Oleurosid	6.2

**Table 2:** Oligonucleotide primer sequences, product sizes, and primer concentrations for quantitative real-time PCR. bp: base pairs; conc: concentration.

Primer	Sequence	Manufacturer	Product size (bp)	Conc. (*μ*M)
AMPK (beta subunit)	5′-agtatcacggtggttgctgt-3′ 5′-caaatactgtgcctgcctct-3′	Biomol Hamburg, Germany	190	0.1
B2M	5′-ggcctgtatgctatccagaa-3′ 5′-gaaagaccagtccttgctga-3′	Biomol, Hamburg, Germany	198	0.4
CI	5′acctgtaaggaccgagaga-3′ 5′-gcaccacaaacacatcaaaa-3′	Biomol, Hamburg, Germany	227	0.1
CIV	5′-ctgttccattcgctgctatt-3′ 5′-gcgaacagcactagcaaaat-3′	Biomol, Hamburg, Germany	217	0.1
CS	5′-aacaagccagacattgatgc-3′ 5′-atgaggtcctgctttgtcct-3′	Biomol, Hamburg, Germany	184	0.1
GAP43	5′ agggagatggctctgctact-3′ 5′ gaggacggggagttatcagt-3′	Biomol Hamburg, Germany	190	0.15
GPx-1	5′-gtccagcgtgtatgccttct-3′ 5′-ctcctggtgtccgaactgat-3′	Biomol, Hamburg, Germany	217	0.1
PGK1	5′-gcagattgtttggaatggtc-3′ 5′-tgctcacatggctgacttta-3′	Biomol, Hamburg, Germany	185	0.4
Sirt1	5′-gtgagaaaatgctggcctaa-3′ 5′-ctgccacaggaactagagga-3′	Biomol, Hamburg, Germany	161	1
SOD2	5′-acagcgcatactctgtgtga-3′ 5′-gggggaacaactcaactttt-3′	Biomol, Hamburg, Germany	183	0.1
Synaptophysin 1	5′-tttgtggttgttgagttcct-3′ 5′-gcatttcctccccaaagtat-3′	Biomol, Hamburg, Germany	204	0.1

**Table 3:** Basal ATP level and ATP concentrations after incubation for 24 h in SY5Y-Mock cells with POS of different concentrations (0.001 nM–10 *μ*M) and after insult with rotenone (250 nM) in SH-SY5Y-Mock cells; basal ATP concentrations served as control, *n* = 7, mean ± SEM, one-way ANOVA with Bonferroni posttest; ^∗^ *P* < 0.05, ^∗∗^ *P* < 0.01, ^∗∗∗^ *P* < 0.001.

Correlated values	ATP (% of control)	ATP after insult (% of control)
0.001 nM vs. control	102.6 ± 2.9	66.9 ± 1.2
0.1 nM vs. control	110.6^∗^ ± 2.0	70.6^∗^ ± 1.5
10 nM vs. control	114.9^∗∗∗^ ± 3.3	70.3^∗^ ± 2.56.1
1 *μ*M vs. control	111.3^∗∗^ ± 1.8	70.4^∗^ ± 2.4
10 *μ*M vs. control	103.4 ± 1.7	67.1 ± 1.6

**Table 4:** Relative normalized mRNA expression levels in brain homogenate from aged and aged POS-treated mice determined using quantitative real-time PCR in comparison to young control animals; mRNA expression of young control mice is 100%; *n* = 9; mean ± SEM with one-way ANOVA and Bonferroni posttest with ^∗^ *P* < 0.05; ^∗∗^ *P* < 0.01; ^∗∗∗^ *P* < 0.001; results are normalized to the mRNA expression levels of beta 2 microglobulin (B2M) and phosphoglycerate kinase 1 (PGK1).

	Aged	Aged + POS
AMP-activated protein kinase (beta subunit)	66.19^∗^ ± 6.00	72.63 ± 8.38
cAMP response binding protein 1 (CREB1)	64.27^∗∗^ ± 4.29	63.28^∗∗^ ± 5.94
Citrate synthase (CS)	66.75^∗^ ± 4.92	61.35^∗∗∗^ ± 9.16
Complex I (CI)	75.16^∗^ ± 3.34	67.53^∗∗^ ± 7.60
Complex IV (CIV)	58.89^∗∗^ ± 5.80	60.97^∗∗^ ± 8.00
Glutathione peroxidase 1 (GPx-1)	67.65^∗^ ± 8.76	58.78^∗∗^ ± 8.18
Growth-associated protein 43 (GAP43)	63.43^∗^ ± 9.23	56.73^∗∗^ ± 8.13
Sirtuin 1 (Sirt1)	74.71^∗^ ± 4.65	75.36^∗∗^ ± 5.99
Superoxide dismutase 2 (SOD2)	97.23 ± 5.26	88.46 ± 10.39
Synaptophysin 1 (SYP1)	83.23 ± 6.08	72.26^∗^ ± 10.43

**Table 5:** Activities of the respiratory chain complexes I and IV in isolated mitochondria of young, aged, and aged mice fed with POS determined using an Oxygraph-2k; *n* = 10, mean ± SEM. GPx-1 was measured in brain homogenate using a calorimetric kit; *n* = 6 ± SEM. CS activity was measured in isolated mitochondria; *n* = 10 ± SEM.

Correlated values	CI activity [(pmol/s^∗^IU CS)]	CIV activity [(pmol/s^∗^IU CS)]	GPx-1 activity (mU/mL)	CS activity (IU/mg protein)
Young vs. aged	1545 ± 101 vs. 1625 ± 80	5487 ± 157 vs. 5673 ± 191	1139 ± 50 vs. 1032 ± 48	927 ± 108 vs. 898 ± 104
Aged vs. aged + POS	1625 ± 80 vs. 1587 ± 108	5673 ± 191 vs. 5806 ± 145	1032 ± 48 vs. 1116 ± 60	898 ± 104 vs. 843 ± 118

## Data Availability

The data used to support the findings of this study are available from the corresponding author upon request.

## Supplementary Materials

Supplementary Materials The supplementary materials include 11 tables showing the complete statistical results including *F* value, degrees of freedom, and *P* value. Supplementary Table 3a: complete statistical results including *F* value, degrees of freedom, and *P* value of number of entries in Y-Maze spontaneous alternation test of young, aged, and POS-treated mice. Supplementary Table 3b: complete statistical results including *F* value, degrees of freedom, and *P* value of alternation rate in Y-Maze spontaneous alternation test of young, aged, and POS-treated mice. Supplementary Table 4: complete statistical results including *F* value, degrees of freedom, and *P* value of number of entries in Passive Avoidance test of young, aged, and POS-treated mice. Supplementary Table 5a: complete statistical results including *F* value, degrees of freedom, and *P* value of basal ATP level in DBCs of young, aged, and POS-treated mice. Supplementary Table 5b: complete statistical results including *F* value, degrees of freedom, and *P* value of ATP concentration in DBCs of young, aged, and POS-treated mice after insult with sodium nitroprusside. Supplementary Table 6a: complete statistical results including *F* value, degrees of freedom, and *P* value of basal ATP concentrations in SY5Y-Mock cells after incubation with POS. Supplementary Table 6b: complete statistical results including *F* value, degrees of freedom, and *P* value of ATP concentration in SY5Y-Mock cells after insult with rotenone. Supplementary Table 7a: complete statistical results including *F* value, degrees of freedom, and *P* value of complex I activity in isolated brain mitochondria of young, aged, and POS-treated mice. Supplementary Table 7b: complete statistical results including *F* value, degrees of freedom, and *P* value of complex IV activity in isolated brain mitochondria of young, aged, and POS-treated mice. Supplementary Table 7c: complete statistical results including *F* value, degrees of freedom, and *P* value of GPx-1 activity in brain homogenate of young, aged, and POS-treated mice. Supplementary Table 7d: complete statistical results including *F* value, degrees of freedom, and *P* value of citrate synthase activity in isolated brain mitochondria of young, aged, and POS-treated mice.
