# Supplementary material for: Effects of Long-Term Treatment with a Blend of Highly Purified Olive Secoiridoids on Cognition and Brain ATP Levels in Aged NMRI Mice
Source: Oxid Med Cell Longev. 2018 Oct 30;2018:4070935. doi: 10.1155/2018/4070935 (PMC6232801; doi:10.1155/2018/4070935)
Supplement: Supplementary Materials — The supplementary materials include 11 tables showing the complete statistical results including F value, degrees of freedom, and P value. Supplementary Table 3a: complete statistical results including F value, degrees of freedom, and P value of number of entries in Y-Maze spontaneous alternation test of young, aged, and POS-treated mice. Supplementary Table 3b: complete statistical results including F value, degrees of freedom, and P value of alternation rate in Y-Maze spontaneous alternation test of young, aged, and POS-treated mice. Supplementary Table 4: complete statistical results including F value, degrees of freedom, and P value of number of entries in Passive Avoidance test of young, aged, and POS-treated mice. Supplementary Table 5a: complete statistical results including F value, degrees of freedom, and P value of basal ATP level in DBCs of young, aged, and POS-treated mice. Supplementary Table 5b: complete statistical results including F value, degrees of freedom, and P value of ATP concentration in DBCs of young, aged, and POS-treated mice after insult with sodium nitroprusside. Supplementary Table 6a: complete statistical results including F value, degrees of freedom, and P value of basal ATP concentrations in SY5Y-Mock cells after incubation with POS. Supplementary Table 6b: complete statistical results including F value, degrees of freedom, and P value of ATP concentration in SY5Y-Mock cells after insult with rotenone. Supplementary Table 7a: complete statistical results including F value, degrees of freedom, and P value of complex I activity in isolated brain mitochondria of young, aged, and POS-treated mice. Supplementary Table 7b: complete statistical results including F value, degrees of freedom, and P value of complex IV activity in isolated brain mitochondria of young, aged, and POS-treated mice. Supplementary Table 7c: complete statistical results including F value, degrees of freedom, and P value of GPx-1 activity in brain homogenate of young [file 4070935.f1.docx]

**Supplementary materials**

**Table 3a**)

| Correlated value | F value | degrees of freedom | P value |
| --- | --- | --- | --- |
| Young vs. aged | 3.813 | 46 | 0.0297 |

**Table 3b)**

| Correlated value | F value | degrees of freedom | P value |
| --- | --- | --- | --- |
| Young vs. 50 % | 80 | 13 | 0.0004 |
| aged vs. 50 % | 120 | 15 | 0.1573 |
| Aged+POS vs. 50 % | 114 | 12 | 0.0058 |

**Table 4)**

| Correlated value | F value | degrees of freedom | P value |
| --- | --- | --- | --- |
| \| \| young vs. aged (day 1) \| \| --- \| \| young vs. aged + POS (day 1) \| \| aged vs. aged + POS (day 1)   \| young vs. Aged (day 2) \| \| --- \| \| young vs. aged + POS (day 2) \| \| aged vs. aged + POS (day 2) \| \| \| \| --- \| --- \| --- \| --- \| --- \| --- \| --- \| \|  \| | 1.29  1.142 | 45  45  45  44  44  44 | \| 0,3177 \| \| --- \| \| 0,9714 \| \| 0,4294 \|  \| 0,3088 \| \| --- \| \| 0,5713 \| \| 0,9006 \| |

**Table 5a)**

| Correlated value | F value | degrees of freedom | P value |
| --- | --- | --- | --- |
| Young vs. aged | 5.334 | 20 | 0.0233 |
| aged vs. aged+POS | 5,334 | 20 | 0.0272 |

**Table 5b)**

| Correlated value | F value | degrees of freedom | P value |
| --- | --- | --- | --- |
| Young vs. aged | 3.912 | 24 | 0.0071 |
| aged vs. aged+POS | 3.912 | 24 | 0.00542 |

**Table 6a)**

| Correlated value | F value | degrees of freedom | P value |
| --- | --- | --- | --- |
| 0,1 nM vs. Medium | 7.083 | 43 | 0.017 |
| 1 μM vs. Medium | 7.083 | 43 | 0.0082 |
| 10 nM vs. Medium | 7.083 | 43 | 0.0005 |

**Table 6b)**

| Correlated value | F value | degrees of freedom | P value |
| --- | --- | --- | --- |
| Rotenon vs. 0,1 nM | 3.526 | 35 | 0.0291 |
| Rotenon vs. 10 nM | 3.526 | 35 | 0.0258 |
| Rotenon vs. 1 μM | 3.526 | 35 | 0.0225 |

**Table 7a)**

**Complex I Activity**

| Correlated value | F value | degrees of freedom | P value |
| --- | --- | --- | --- |
| young vs. aged | 0.5223 | 27 | 0.6171 |
| Aged vs. aged+POS | 0.5223 | 27 | 0.9873 |

**Table 7b)**

**Complex IV Activity**

| Correlated value | F value | degrees of freedom | P value |
| --- | --- | --- | --- |
| young vs. aged | 0.9018 | 28 | 0.7177 |
| Aged vs. aged+POS | 0.9018 | 28 | 0.8350 |

**Table 7c)**

**GPx-1 Activity**

| Correlated value | F value | degrees of freedom | P value |
| --- | --- | --- | --- |
| young vs. aged | 1.018 | 15 | 0.3964 |
| Aged vs. aged+POS | 1.018 | 15 | 0.5259 |

**Table 7d)**

**CS Activity**

| Correlated value | F value | degrees of freedom | P value |
| --- | --- | --- | --- |
| young vs. aged | 0,1441 | 23 | 0,8647 |
| Aged vs. aged+POS | 0,1441 | 23 | 0,9302 |
